# Efficacy and safety of efgartigimod PH20 SC for Sjögren’s disease-associated dryness: study protocol for an investigator-initiated, multicenter, phase 2, randomized, double-blind, placebo-controlled trial (OASIS study)

**DOI:** 10.3389/fmed.2025.1719757

**Published:** 2026-01-02

**Authors:** Yushiro Endo, Naoki Hosogaya, Yuri Fukushige, Sawana Narita, Julie Jacobs, William Reiss, Yuya Imai, Toshimasa Shimizu, Tomohiro Koga, Hiroshi Yamamoto, Tomoya Sakai, Yukinori Takagi, Misa Sumi, Atsushi Kawakami

**Affiliations:** 1 Department of Immunology and Rheumatology, Division of Advanced Preventive Medical Sciences, Nagasaki University Graduate School of Medical Sciences, Nagasaki, Japan; 2 Clinical Research Center, Nagasaki University Hospital, Nagasaki, Japan; 3 Department of Hospital Pharmacy, Nagasaki University Hospital, Nagasaki, Japan; 4 Argenx, Ghent, Belgium; 5 Department of Integrated Science and Technology, Graduate School of Integrated Science and Technology, Nagasaki University, Nagasaki, Japan; 6 Department of Radiology and Biomedical Informatics, Nagasaki University Graduate School of Biomedical Sciences, Nagasaki, Japan

**Keywords:** Sjögren’s disease, dryness, FcRn, efgartigimod, ESSPRI

## Abstract

**Background:**

Sjögren’s disease (SjD) is a chronic systemic autoimmune disease characterized by lymphocytic infiltration and progressive destruction of the lacrimal and salivary glands, leading to ocular and oral dryness as hallmark symptoms. Despite low systemic activity, these sicca symptoms significantly impair the quality of life, particularly in patients with severe dryness. Currently, no disease-modifying therapy is approved for SjD. Efgartigimod PH20, the neonatal Fc receptor (FcRn) blocker, has shown promising efficacy and safety in patients with moderate-to-severe systemic SjD. However, its efficacy for dryness in patients with SjD remains unknown.

**Methods/design:**

This is a phase 2, multicenter, randomized, double-blind, placebo-controlled, investigator-initiated trial conducted in Japan. Approximately 45 adult patients with SjD and moderate-to-severe dryness will be randomized in a 1:1:1 ratio to receive subcutaneous efgartigimod PH20 at 1,000 mg weekly (QW), 1,000 mg every other week (Q2W), or placebo for 24 weeks. The primary endpoint is the change in EULAR Sjögren’s Syndrome Patient-Reported Index (ESSPRI)-dryness score from baseline to week 24. Key secondary endpoints include changes in ESSPRI-total, ESSPRI-fatigue, Diary of Sjögren’s Symptoms Assessment (DiSSA) scores, and the proportion of responders in ESSPRI and Sjögren’s Tool for Assessing Response (STAR) assessments. After the blinded period, all participants will be offered an open-label extension treatment to assess long-term safety and efficacy.

**Discussion:**

This trial specifically targets patients with SjD who have prominent sicca symptoms. By using FcRn blockade to reduce pathogenic IgG autoantibodies, this study aims to explore the potential of efgartigimod PH20 as a novel therapeutic approach for dryness-predominant SjD. The findings are expected to provide future treatment strategies to address the major unmet need regarding dryness-related burden in this patient population.

**Clinical trial registration:**

Japan Registry of Clinical Trials (jRCT2071250042); registered on 08 July 2025, https://jrct.mhlw.go.jp/en-latest-detail/jRCT2071250042.

## Background

Sjögren’s disease (SjD) is a systemic autoimmune disease characterized by lymphocytic infiltration and progressive destruction of the lacrimal and salivary glands, leading to ocular and oral dryness as the hallmark symptoms. Although the disease can extend beyond the exocrine glands and cause systemic inflammation with extra-glandular manifestations (1), life-threatening systemic involvement occurs in only a minority of patients (approximately 13%) (2). In contrast, the majority of patients suffer from sicca symptoms—dry eye and/or dry mouth (3), which substantially impact their quality of life (QOL) by interfering with daily activities such as speaking, swallowing, and eating (4, 5). Salivary gland dysfunction can lead to dysphagia, oral pain, burning sensations, and oral infections, while tear deficiency causes photosensitivity, eye pain, foreign body sensation, corneal ulcers, and ocular infections. In addition to such sicca-related burden (6), QOL-related general manifestations, including fatigue (7), sleep disturbances (8), and generalized pain (9), are frequently observed in patients with SjD.

The reported prevalence of SjD varies widely across studies, ranging from 0.013% to as high as 3.5% (10–13). Despite its prevalence and burden, there are no approved disease-modifying treatments targeting SjD. Current treatment options are limited to symptomatic management with artificial saliva and tears, salivary stimulants, and procedures, such as punctal occlusion or cauterization, highlighting a critical unmet need for effective treatments targeting SjD.

Efgartigimod, a human Fc fragment engineered to block the neonatal Fc receptor (FcRn), has offered a novel therapeutic approach for autoimmune diseases including myasthenia gravis, immune thrombocytopenia, and chronic inflammatory demyelinating polyneuropathy by selectively reducing levels of circulating IgG including the pathogenetic autoantibodies (14, 15). Efgartigimod does not affect other immunoglobulins that are not recycled by FcRn or albumin, ensuring a targeted mechanism with minimal off-target effects (16). Efgartigimod is expected to attenuate SjD-related symptoms by reducing levels of the pathogenetic autoantibodies such as anti-SSA/Ro antibody, which contribute to the pathogenesis of SjD (17). The completed phase 2 study (NCT05817669) in participants with moderate-to-severe systemic SjD (EULAR Sjögren’s Syndrome Disease Activity Index; ESSDAI ≥ 5) demonstrated that efgartigimod was safe and well tolerated, the pharmacodynamic (PD) effects were as expected (approximately 60% mean reduction in total IgG), and based on totality of data, signals for potential efficacy were observed (the proportion of responders to the Composite of Relevant Endpoints for Sjögren’s Syndrome as the primary endpoint; efgartigimod group, 45.5% vs. the placebo group, 11.1%) (18).^1^ A phase 3 study (NCT06684847; jRCT2031240584) is ongoing in participants with moderate-to-severe systemic SjD (clinical ESSDAI (clinESSDAI) ≥ 6).^2^

Guided by these findings, the current investigator-initiated trial was designed to evaluate subcutaneous efgartigimod in patients with moderate-to-severe dryness symptoms of SjD.^3^ This phase 2 study is focused on patients with prominent sicca symptoms (EULAR Sjögren’s Syndrome Patient-Reported Index-dryness; ESSPRI-dryness ≥ 5) but low systemic activity (clinESSDAI < 6), a population representing a major unmet need for the treatment of SjD. By targeting the patient-reported dryness domain as the primary endpoint, the study aims to determine whether FcRn blockade can provide meaningful clinical benefits for SjD-associated dryness.

## Methods/design

### Study design

This study is conducted according to the protocol and the following: Consensus ethical principles derived from international guidelines, including the Declaration of Helsinki and the International Ethical Guidelines of the Council for International Organizations of Medical Sciences; the applicable International Council for Harmonization of Technical Requirements for Pharmaceuticals for Human Use (ICH)/Good Clinical Practice (GCP) guidelines; and all applicable laws and regulations.

This is a phase 2, randomized, double-blinded, placebo-controlled, parallel-group, three-arm, multicenter, Japanese investigator-initiated study evaluating treatment with efgartigimod with recombinant human hyaluronidase PH20 (rHuPH20), administered subcutaneously (“efgartigimod PH20 SC”) every week (QW) or once every 2 weeks (Q2W), compared to placebo for 24 weeks in participants with SjD-associated moderate-to-severe dryness symptoms based on ESSPRI-total ≥ 5, ESSPRI-dryness ≥ 5, and clinESSDAI < 6. This study aims to evaluate the efficacy, safety, tolerability, pharmacokinetic (PK), and pharmacodynamic (PD) of efgartigimod PH20 SC compared to placebo in participants with SjD in Japan. QW and Q2W regimens were chosen based on prior studies demonstrating sustained IgG suppression and tolerability across autoimmune indications, including systemic SjD (19). With this study, we aim to further investigate both dosing regimens in patients with low systemic disease activity and at least moderate dryness symptoms. The study will be conducted at 15 centers in Japan. The local ethics committee at each center will approve the study (approval is already in place in Nagasaki University Hospital; Approval No. I25-601).

**Figure 1:**
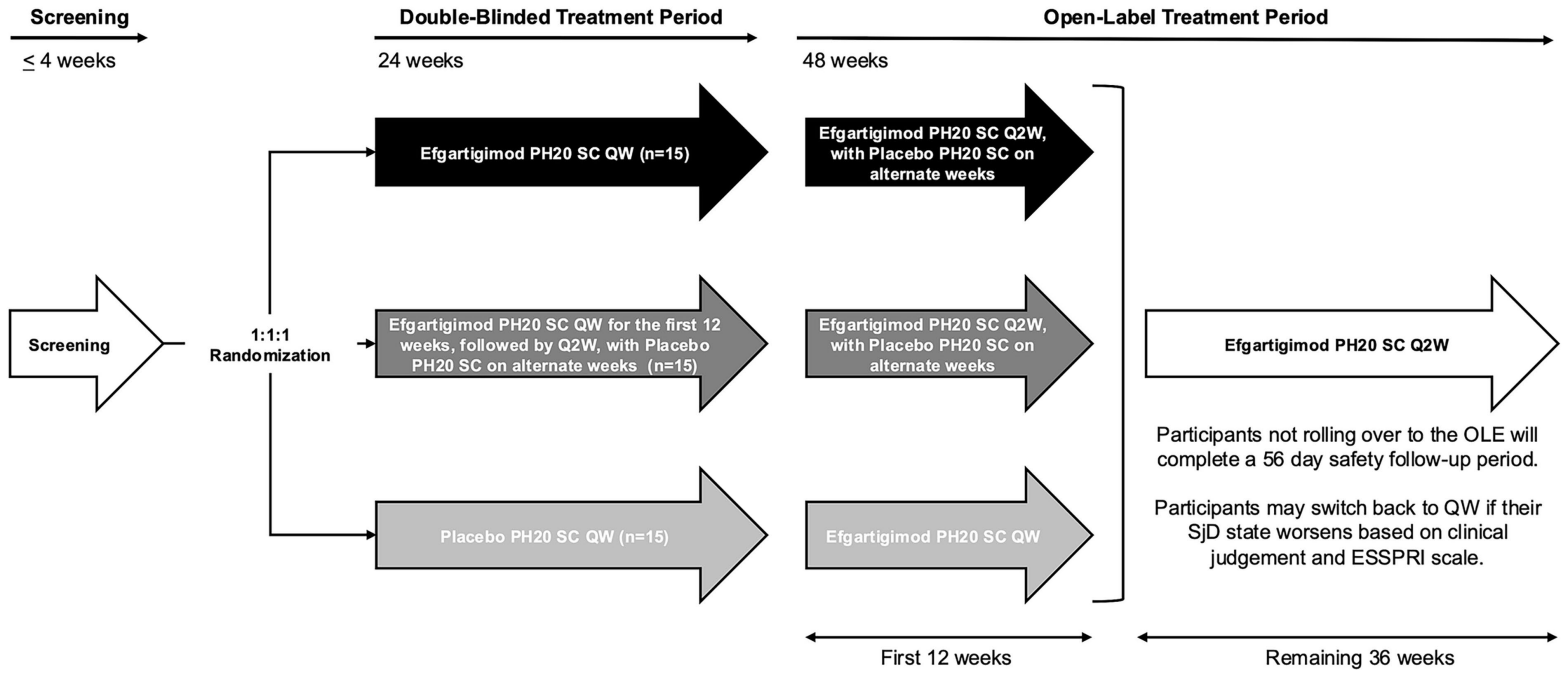
Schema of the study design. Efgartigimod PH20 SC, efgartigimod for SC administration coformulated with recombinant human hyaluronidase PH20; ESSPRI, EULAR Sjögren’s Syndrome Patient-Reported Index; EULAR, European Alliance of Associations for Rheumatology; *n*, number of participants per arm; OLE, open-label extension; placebo PH20 SC, placebo; PH20, hyaluronidase; QW, every week; Q2W, once every 2 weeks; SC, subcutaneous(ly); SjD, Sjögren’s disease.

**Figure 2:**
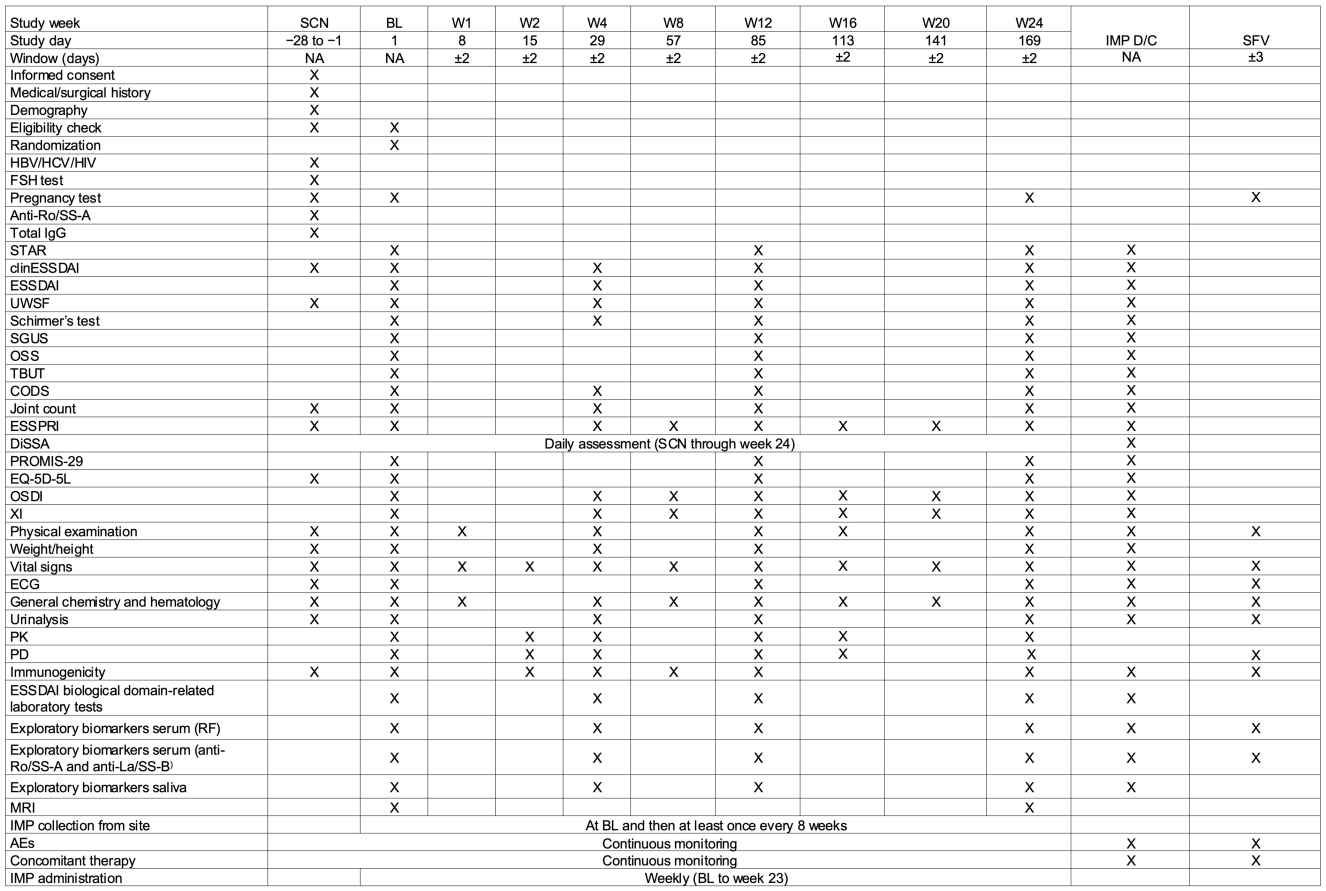
Schedule of activities: double-blinded treatment period. AE, adverse event; BL, baseline; clinESSDAI, clinical EULAR Sjögren Syndrome Disease Activity Index; CODS, clinical oral dryness score; D/C, discontinuation; DiSSA, Diary of Sjögren’s Symptoms Assessment; ECG, electrocardiogram; ESSDAI, EULAR Sjögren’s Syndrome Disease Activity Index; ESSPRI, EULAR Sjögren’s Syndrome Patient-Reported Index; EULAR, European Alliance of Associations for Rheumatology; FSH, follicle-stimulating hormone; HBV, hepatitis B virus; HCP, health care professional; HCV, hepatitis C virus; IgG, immunoglobulin G; IMP, investigational medicinal product; MRI, magnetic resonance imaging; NA, not applicable; OSDI, Ocular Surface Disease Index; OSS, ocular staining score; PD, pharmacodynamic(s); PK, pharmacokinetic(s); PROMIS-29, Patient-Reported Outcomes Measurement Information System 29; RF, rheumatoid factor; rHuPH20, recombinant human hyaluronidase PH20; SCN, screening; SFV, safety follow-up visit; SGUS, salivary gland ultrasonography; SS-A, Sjögren’s syndrome–related antigen A; SS-B, Sjögren’s syndrome–related antigen B; STAR, Sjögren’s Tool for Assessing Response; TBUT, tear break-up time; UWSF, unstimulated whole salivary flow; W, week; XI, xerostomia inventory.

All participants’ eligibility is confirmed by the investigator at both screening and baseline. During the double-blind treatment period, the investigator, the study team, and participants are blinded to the treatment arm. During the open-label extension (OLE) treatment period, all participants and site staff are aware of receiving efgartigimod PH20 SC. The study design is summarized in Figure 1. Briefly, this study is divided into the following periods: Screening period (≤4 weeks): Participants will have a baseline visit within 4 weeks of the screening visit. Double-blinded treatment period (24 weeks): All participants will be randomized using permuted block randomization using interactive response technology (IRT) in a 1:1:1 ratio to receive efgartigimod PH20 SC 1,000 mg QW, efgartigimod PH20 SC Q2W (receiving efgartigimod PH20 SC QW for the first 12 weeks, followed by efgartigimod PH20 SC Q2W with placebo on alternate weeks), or placebo QW, respectively. Following randomization, participants will enter a 24-week double-blinded treatment period, during which IMP will be injected subcutaneously every week starting on day 1 (baseline) and continuing through week 23. At the end of the double-blinded treatment period (week 24), participants will be given the option to roll over into an OLE treatment period (48 weeks), which will assess the long-term safety, tolerability, and efficacy of efgartigimod PH20 SC. For the first 12 weeks of the OLE, participants who received efgartigimod PH20 SC QW in the double-blinded treatment period will receive efgartigimod PH20 SC Q2W (receiving placebo on alternate weeks). Participants who received efgartigimod PH20 SC Q2W in the last 12 weeks of the double-blinded treatment period will continue to receive efgartigimod PH20 SC Q2W (receiving placebo on alternate weeks). Participants who received placebo QW in the double-blinded treatment period will receive efgartigimod PH20 SC QW. After the first 12 weeks of the OLE, all participants will receive efgartigimod PH20 SC Q2W. If a participant’s disease state worsens while on Q2W dosing, as determined by the investigator’s clinical judgment and the ESSPRI scale, they may switch back to QW dosing. All participants and site staff will remain blinded to their initial treatment assignment throughout the study. The schedule of activities for the double-blinded treatment period is summarized in Figure 2.

### Participant recruitment

Participants will be recruited at 15 sites in Japan, including Nagasaki University Hospital, Kanazawa Medical University Hospital, NHO Tokyo Medical Center, Hamamatsu University Hospital, and Hyogo Medical University Hospital. All eligible patients will be selected and approached based on information derived from the electronic health records of these hospitals according to the inclusion and exclusion criteria. Participants will be provided with an explanation of the study from their treating rheumatologists and the clinical research coordinator (CRC), and they will be asked to voluntarily sign an informed consent before participation. Although participation is not restricted by ethnicity, all participants in this study are expected to be of Japanese descent, as the trial is conducted exclusively at sites in Japan.

#### Inclusion criteria

Participants are eligible for the study only if they fulfill all of the following criteria.

1. Is at least 18 years of age and meets the local legal age of consent for clinical studies when signing the informed consent form (ICF).
2. Has a medical history of fulfilling the 2016 American College of Rheumatology (ACR)/European Alliance of Associations for Rheumatology (EULAR) classification criteria for SjD (20).
3. Has a clinESSDAI < 6 at screening.
4. Has ESSPRI-total ≥ 5 and ESSPRI-dryness ≥ 5 at screening
5. Is anti-Ro/SS-A positive in the central laboratory at screening
6. Has a residual unstimulated whole salivary flow (UWSF) ≥ 0.01 mL/min at screening.
7. Has provided signed informed consent and agrees to comply with protocol requirements.
8. Is a woman(en) of childbearing potential (WOCBP) who agrees to use contraceptive measures consistent with local regulations and study requirements.
9. Is a WOCBP who has a negative serum pregnancy test at screening and a negative urine pregnancy test at baseline before receiving an investigational medicinal product (IMP).

#### Exclusion criteria

Participants are excluded from the study if any of the following criteria apply:

1. Has a known autoimmune disease or any medical condition, besides the indication under study, that would interfere with an accurate assessment of clinical symptoms of SjD or put the participant at undue risk
2. Has active fibromyalgia that is not adequately controlled in the investigator’s clinical judgment or is receiving fibromyalgia treatment that has not been stable for at least 12 weeks before screening
3. Has any severe systemic SjD manifestation that is not adequately controlled at baseline and that may put the participant at undue risk based on the investigator’s clinical judgment
4. Has secondary (also referred to as Associated) Sjögren’s disease, defined as an overlap with another autoimmune rheumatic or systemic inflammatory condition (e.g., rheumatoid arthritis, systemic lupus erythematosus, scleroderma, or idiopathic inflammatory myopathy)
5. Has severe renal impairment with an estimated glomerular filtration rate < 30 mL/min/1.73 m^2^ at screening
6. Has a history of malignancy unless considered cured by adequate treatment with no evidence of recurrence for ≥3 years before the first IMP administration. Adequately treated participants with the following cancers can be included at any time:

  a.  Basal cell or squamous cell skin cancer
  b.  Carcinoma *in situ* of the cervix
  c.  Carcinoma *in situ* of the breast
  d.  Incidental histological findings of prostate cancer (TNM stage T1a or T1b).

7. Has a severe or serious active infection that is not sufficiently resolved before baseline OR an active infection that would put the participant at undue risk OR would confound the study results in the investigator’s judgment
8. Has a positive serum test result at screening for active infection with any of the following:

  a.  HBV is indicative of an acute or chronic infection unless associated with a negative HBV DNA test result
  b.  HCV based on an HCV antibody assay unless a negative RNA test result is available
  c.  HIV based on test results of a CD4 count < 200 cells/mm^3^
  d.  Having an AIDS-defining condition
  e.  HIV based on test results of a CD4 count ≥ 200 cells/mm^3^, but not adequately treated with antiviral therapy.

9. Has a disease or any other medical condition that, in the investigator’s clinical judgment, would confound the study results or put the participants at undue risk, which includes recent major surgery (within 3 months of screening) or intention to have major surgery during the study
10. Has a history (within 12 months before screening) of current alcohol, drug, or medication abuse as assessed by the investigator. Any use of cannabis or cannabinol, recreational or prescribed, is prohibited.
11. Is pregnant or lactating or intention to become pregnant during the study
12. Has a total IgG concentration of < 4 g/L at screening
13. Has received IVIg, SCIg, or PLEX received <4 weeks before and during the screening period
14. Has live or live-attenuated vaccine received <4 weeks before and during the screening period
15. Has used pilocarpine and/or any other pharmacological stimulant for the salivary and lacrimal glands not at a stable dose for at least 4 weeks before the screening period, or has started ≤ 4 weeks before the screening
16. Has chronic use of topical immunosuppressive ophthalmic agents (e.g., cyclosporine) < 4 weeks before and during the screening period
17. Has chronic use (more than 4 weeks) of anticholinergic agents (e.g., tricyclic antidepressants, antihistamines, phenothiazines, antiparkinsonian drugs, or anti-asthmatic medications) that cause xerostomia/dry eyes, unless these medications are at a stable dose for ≥4 weeks before screening, with a dose regimen stable during the screening period

18. Has corticosteroid use:

  a.  Systemic corticosteroids ≤ 4 weeks before and during the screening period
  b.  Topical corticosteroids for current SjD-related condition or medical history ≤1 week before screening
  c.  Local corticosteroid injections ≤ 4 weeks before and during the screening period (for corticosteroid irrigation of the salivary gland ≤8 weeks before and during the screening period)
  d.  Ophthalmic corticosteroids ≤ 1 week before and during the screening period.

19. Has used cyclophosphamide < 6 months before and during the screening period
20. Has used JAK inhibitors or TYK2 inhibitors ≤ 4 weeks before and during the screening period
21. Has used DMARDs and antimalarials ≤ 3 months before randomization
22. Has received or are receiving the prior and concomitant therapies without their respective washout periods (cyclophosphamide, monoclonal antibodies or other biologic DMARDs, JAK inhibitors/TYK2 inhibitors, PLEX or immunoadsorption, any other experimental IMP, immunoglobulin therapy, and live or live-attenuated vaccines)
23. Has a monoclonal antibody that is not an anti-CD20 nor an anti-CD19 nor an Fc-fusion protein received < 4 weeks or < 5 half-lives before screening, whichever is longer
24. Has used opioid medications during screening
25. Has received an anti-CD20 or anti-CD19 antibody < 6 months before and during the screening period
26. Is currently participating in another interventional study (participation in observational studies is permitted)
27. Has received different IMP in another clinical study < 12 weeks or < 5 half-lives (whichever is longer) before screening
28. Has known hypersensitivity to IMP or 1 of its excipients
29. Has previous participation in an efgartigimod clinical study and received at least 1 dose of IMP.

## Randomization and blinding

Randomization is performed centrally using an IRT system applying permuted block randomization (1:1:1 ratio). Allocation concealment is ensured via IRT, with access restricted to unblinded personnel not involved in assessments. The translational Research Informatics Center (TRI) independently oversees these procedures.

### Adverse events

Adverse events (AEs) will be reported by the participant (or, if appropriate, by the caregiver or surrogate). The investigator and qualified designees are responsible for detecting, documenting, and recording events that meet the definition of an AE or serious adverse event (SAE) and monitoring all reported events, including those reported by the participant. All AEs will be collected from signing the informed consent form (ICF) until the safety follow-up visit (SFV). All serious adverse events (SAEs) will be recorded and reported to the sponsor-investigator or designee immediately, and under no circumstance will this exceed 24 h. After the initial AE/SAE report, the investigator must proactively follow up with each participant at subsequent visits/contacts. All SAEs will be followed up until resolution stabilization, the event is otherwise explained, or the participant is lost to follow-up.

### Efficacy assessments

All the endpoints and objectives in this study are summarized in Table 1. Briefly, the primary endpoint is the change from baseline in ESSPRI-dryness at week 24 to evaluate the efficacy of efgartigimod PH20 SC administered QW or Q2W compared to placebo on dryness. Diary of Sjögren’s Symptoms Assessment (DiSSA) is an SjD-specific symptom severity diary developed for the ongoing phase 3 study (NCT06684847; jRCT2031240584) to assess symptom severity in participants with SjD. Although objective ocular indicators are not part of the inclusion criteria, ocular assessments, including Schirmer’s test, ocular staining score (OSS), tear break-up time (TBUT), and ocular surface disease index (OSDI), are evaluated as secondary endpoints throughout the study. The key secondary endpoint is to evaluate the efficacy of efgartigimod PH20 SC administered QW or Q2W compared to placebo on patient-reported outcomes (PROs) and Sjögren’s Tool for Assessing Response (STAR). This composite measure incorporates patient-reported outcome (ESSPRI) with functional tests (e.g., UWSF, Schirmer’s test, OSS, and SGUS) and systemic activity indices (ClinESSDAI) (21), enabling a holistic assessment of treatment response in SjD.

**Table 1:** Objectives and endpoints of the study.

Objectives	Endpoints
Primary endpoints	
To evaluate the efficacy of efgartigimod PH20 SC administered QW or Q2W compared to the placebo at week 24 on dryness	Change from baseline in ESSPRI-dryness at week 24
Key secondary endpoints	
To evaluate the efficacy of efgartigimod PH20 SC QW or Q2W compared to placebo on PROs and a composite measure	Change from baseline in ESSPRI-total score at week 24 Change from baseline in ESSPRI-fatigue score at week 24 Change from baseline in ESSPRI-pain score at week 24 Proportion of responders (improvement in ESSPRI-total score >1.0 point/15%) at week 24 The proportion of responders in STAR at week 24 Change from baseline in DiSSA total score at week 24 Change from baseline in DiSSA joint pain at week 24 Change from baseline in DiSSA fatigue at week 24 Change from baseline in DiSSA dryness at week 24
Other secondary endpoints	
To evaluate the efficacy of efgartigimod PH20 SC QW or Q2W on glandular function and overall disease activity compared to placebo	Change from baseline in clinESSDAI at week 24 Change from baseline in ESSDAI at week 24 Change from baseline in UWSF at week 24 Change from baseline in Schirmer’s test at week 24 Change from baseline in SGUS at week 24 Change from baseline in OSS at week 24 Change from baseline in TBUT at week 24 Change from baseline in CODS at week 24 Change from baseline in joint count at week 24
To evaluate the impact of efgartigimod PH20 SC compared to placebo on health-related quality of life and health status in the treatment of SjD	Change from baseline in PROMIS-29 domain score at week 24 Change from baseline in EQ-5D-5L at week 24 Change from baseline in OSDI at week 24 Change from baseline in XI at week 24
To assess the immunogenicity of efgartigimod PH20 SC	Incidence and prevalence of ADA against efgartigimod in serum Incidence and prevalence of antibodies against rHuPH20 in plasma
To evaluate the PK of efgartigimod PH20 SC	Efgartigimod serum concentrations over time
To assess the PD of efgartigimod PH20 SC QW or Q2W	Total IgG serum concentrations of efgartigimod PH20 SC administered QW and Q2W Percent change from baseline in total IgG levels in serum over time
To assess the safety and tolerability of efgartigimod PH20 SC compared to placebo	Incidence, severity, and relatedness to IMP of AEs, SAEs, and AEs leading to IMP discontinuation Clinically significant changes in laboratory parameters, ECGs, and vital signs

ADA, antidrug antibody(ies); AE, adverse event; clinESSDAI, clinical EULAR Sjögren’s Syndrome Disease Activity Index; CODS, clinical oral dryness score; DiSSA, Diary of Sjögren’s Symptoms Assessment; ECG, electrocardiogram; efgartigimod PH20 SC, efgartigimod for SC administration coformulated with rHuPH20; ESSDAI, EULAR Sjögren’s Syndrome Disease Activity Index; ESSPRI, EULAR Sjögren’s Syndrome Patient-Reported Index; EULAR, European Alliance of Associations for Rheumatology; IgG, immunoglobulin G; IMP, investigational medicinal product; OSDI=Ocular Surface Disease Index; OSS, ocular staining score; PD, pharmacodynamic(s); PH20, hyaluronidase; PK, pharmacokinetic(s); PRO, patient-reported outcome; PROMIS, Patient-Reported Outcome Measurement Information System 29; SjD, Sjögren’s disease; QW, every week; Q2W, every 2 weeks; RF, rheumatoid factor; rHuPH20, recombinant human hyaluronidase PH20; SAE, serious adverse event; SC, subcutaneous(ly); SGUS, salivary gland ultrasonography; STAR, Sjögren’s Tool for Assessing Response; TBUT, tear break-up time; UWSF, unstimulated whole salivary flow; XI, xerostomia inventory.

Key secondary endpoints:

1. Change from baseline in ESSPRI-total score at week 24
2. Change from baseline in ESSPRI-fatigue score at week 24
3. Change from baseline in ESSPRI-pain score at week 24
4. Proportion of responders (improvement in ESSPRI-total score >1.0 point/15%) at week 24
5. The proportion of responders in STAR at week 24
6. Change from baseline in DiSSA total score at week 24
7. Change from baseline in DiSSA joint pain at week 24
8. Change from baseline in DiSSA fatigue at week 24
9. Change from baseline in DiSSA dryness at week 24.

### Safety assessments

Safety assessments (e.g., physical examination, vital signs, electrocardiograms, laboratory tests, and pregnancy testing) will be performed before IMP administration. At screening, abnormalities in any safety assessment that confirm a preexisting condition will be reported as a medical history. Safety endpoints include AEs, SAEs, AEs leading to IMP discontinuation, vital signs, electrocardiograms, and clinical laboratory safety evaluations. All safety analyses will be based on the safety analysis set.

#### Sample size considerations

Approximately 45 participants will be randomized, with approximately 15 participants per arm. The sample size has been calculated to achieve minimally acceptable precision to estimate the difference between each of the efgartigimod arms and the placebo arm on the changes from baseline at week 24 in ESSPRI-dryness. It is expected that approximately 13 participants per arm will complete the treatment period, with a standard deviation of approximately 2.00 points. This study is exploratory in nature, and a two-sided significance level of 10% with a 90% CI was adopted to detect potential signals. The proposed sample size is expected to result in a standard error for the estimated mean difference of approximately 0.78 points and a half-width for the 90% CI < 1.34 points. The planned sample size provides approximately 85% power to detect a true difference (efgartigimod vs. placebo) of approximately 2.00 points in the change from baseline in ESSPRI-dryness at week 24 using a two-sided significance level of 10% and Dunnett’s test.

### Statistical analysis

We prepared a statistical analysis plan (SAP), where all details regarding the statistical analysis and the preparation of tables, listings, and figures will be described. Data will be listed and summarized using SAS Version 9.4 (SAS Institute, Inc., Cary, North Carolina, US) or higher.

The following descriptive statistics will be used to summarize the data based on their nature, unless otherwise specified: continuous variables: number of non-missing observations, mean, standard deviation, median, minimum, maximum, and number of missing observations. Categorical variables include frequencies, percentages, and number of missing observations/events.

For all the primary and key secondary endpoint analyses, 90% CIs and *p*-values for comparing treatment groups will be presented unless otherwise specified, along with other inferential statistics as appropriate for the double-blinded treatment period. For the OLE treatment period only, descriptive summaries will be presented, and no inferential analysis will be performed. The baseline value for descriptive analyses of the OLE will either be the baseline of the DB part or will be the last non-missing assessment before the first administration of the IMP, including unscheduled visits during the double-blinded treatment period.

Statistical analyses are performed independently by the TRI.

## Discussion

The primary motivation for this trial is based on the recognition that sicca symptoms (dry eyes and mouth) have a significant impact on the QOL for most patients with SjD. There are no systemic disease-modifying treatments approved for SjD. Randomized trials of potential disease-modifying treatments, which have already been approved for other autoimmune diseases, have not demonstrated clinically significant improvements in sicca symptoms in SjD (22–24). For instance, a placebo-controlled trial of rituximab failed to meet its primary endpoint, which was the proportion of patients achieving a 30% improvement in either oral dryness or fatigue at 48 weeks (22). In addition, hydroxychloroquine did not improve dryness, pain, or fatigue over 24 weeks compared to placebo (23). More recently, a phase 3 randomized study of abatacept did not show significant clinical efficacy compared to placebo (24). These findings highlight the difficulty in modifying sicca symptoms, which may reflect irreversible glandular damage, multifactorial pathophysiology, and the limitations of previously tested agents.

The present investigator-initiated trial specifically targets patients with moderate-to-severe dryness but low systemic activity (clinESSDAI < 6), a subgroup that represents the majority of SjD patients in clinical practice.

While ESSDAI <5 is a well-established threshold for defining low systemic disease activity in SjD (25), our inclusion criteria are based on ClinESSDAI. Owing to differences in domain weighting between the two indices (26), a ClinESSDAI score of 6 was approximately equivalent to an ESSDAI score of 5 based on data from the completed phase 2 study (NCT05817669) in participants with moderate-to-severe systemic SjD (https://clinicaltrials.gov/study/NCT05817669). This approach was chosen to ensure alignment with the ongoing phase III study, including participants with at least moderate systemic disease activity (ClinESSDAI ≥6). (NCT06684847; jRCT2031240584). By enrolling patients with residual salivary flow and high ESSPRI-dryness scores, this study aims to maximize the potential to detect meaningful improvements in sicca symptoms. The trial incorporates a variety of validated PROs, including ESSPRI-dryness as the primary endpoint, complemented by a variety of objective measures focusing on dry eyes and mouth.

One limitation of this study is that, although the inclusion criteria required both ESSPRI-total ≥5 and ESSPRI-dryness ≥5, participants may still present heterogeneous symptom profiles, and in some cases, fatigue or pain may be more prominent than dryness. Therefore, in addition to the total ESSPRI, each ESSPRI domain (dryness, fatigue, and pain) will also be analyzed separately. Multivariable regression and subgroup analyses will be conducted to determine whether baseline symptom profiles modify the treatment effect and to confirm the robustness of efficacy in dryness-predominant populations.

In summary, this trial is designed to bridge a crucial gap in SjD management by focusing on patient-reported dryness as the primary endpoint with a novel immunotherapeutic strategy. This study has the potential to not only introduce a promising therapy for SjD-associated dryness and impaired QOLs but also lay the foundation for future research aimed at improving outcomes in SjD.

## Footnotes

1
https://clinicaltrials.gov/study/NCT05817669

2
https://clinicaltrials.gov/study/NCT06684847

3
https://jrct.mhlw.go.jp/en-latest-detail/jRCT2071250042
